# Detection of SARS-CoV-2 RNA and Biomarkers in Device-Captured Droplets From the Lung

**DOI:** 10.1016/j.chpulm.2025.100137

**Published:** 2025-01-22

**Authors:** Rebecca S. Steinberg, Tzu-Chun Chu, Denny Shin, Binh Ha, He-Ying Sun, Samadhan J. Jadhao, David N. Ku, Blaine R. Roberts, Evan J. Anderson, Laila Hussaini, Larry J. Anderson, Blake Anderson

**Affiliations:** a Department of Medicine, Division of General Medicine, Emory University, Atlanta; b College of Public Health, University of Georgia, Athens; c Division of Infectious Diseases, Department of Pediatrics, Emory University and Children’s Healthcare of Atlanta, Atlanta; d School of Mechanical Engineering, Georgia Institute of Technology, Atlanta; e Department of Biochemistry, Emory University School of Medicine, Atlanta, GA

**Keywords:** cough specimen, COVID-19, lower respiratory tract infection, lung biomarkers

## Abstract

**Background:**

SARS-CoV-2 remains a global health issue since its discovery in 2019, and long-term noninvasive clinical testing methods are required. The current preferred method of detection is nasopharyngeal swab, which reflects sampling of the upper respiratory tract alone.

**Research Question:**

Can we noninvasively assess detection of SARS-CoV-2 RNA and biomarkers in patients’ cough droplets captured with the PneumoniaCheck device?

**Study Design and Methods:**

We enrolled adult patients with a recent nasopharyngeal swab that was positive for COVID-19 by polymerase chain reaction (PCR) who were receiving monoclonal antibody infusion therapy. After consent and instruction, patients coughed 5 sets of 10 coughs into the PneumoniaCheck device. Material captured on the device filter was eluted and tested for biomarkers (interferon gamma, tumor necrosis factor alpha [TNF-α], IL-1β, IL-2, IL-4, IL-6, IL-8, IL-10, IL-12p70, and IL-13) and amylase by an enzyme activity assay, and SARS-CoV-2 RNA by PCR (3 different primer sets).

**Results:**

A total of 44 case patients out of 50 cases and 17 control patients with adequate specimen and accompanying clinical data were included in the analysis. Thirty case patient cough specimens (68%) tested PCR positive for SARS-CoV-2 RNA, 10 (23%) tested negative, and 4 (9%) tested indeterminate. IL-13 and TNF-α levels were significantly higher, whereas IL-2 levels were significantly lower in case specimens than in control cough specimens. In the multivariable analysis of biomarkers and reported symptoms, higher IL-10 levels were associated with reduced fatigue (OR, 0.41; 95% CI, 0.15-0.87; *P* = .039), whereas higher IL-12p70 (OR, 2.74; 95% CI, 1.15-8.51; *P* = .043), IL-4 (OR, 3.56; 95% CI, 1.56-11.20; *P* = .008), and TNF-α (OR, 4.36; 95% CI, 1.79-14.60; *P* = .004) levels were associated with fever.

**Interpretation:**

Our results show that the PneumoniaCheck device is a noninvasive method for successfully detecting SARS-CoV-2 and inflammatory cytokines in specimens from the lower respiratory tract in patients with COVID-19 and likely in patients with other lung diseases.

**Take-Home Points**

**Study Question:** Can the PneumoniaCheck device capture coughed droplets from the lung with detectable levels of SARS-CoV-2 RNA and biomarkers?

**Results:** Most PneumoniaCheck collected specimens contained detectable levels of SARS-CoV-2 RNA and biomarkers with no or minimal upper respiratory contamination.

**Interpretation:** Our results show that the PneumoniaCheck device is a noninvasive method to obtain specimens from the lower respiratory tract for detection of SARS-CoV-2 RNA and biomarkers that can help understand COVID-19 and, likely, other lung disease.

Diagnosis and characterization of lung disease including pneumonia is often hampered by the lack of a lower respiratory tract (LRT) specimen. Sputum is the most common LRT specimen used to diagnose pneumonia but may not be available or may be of poor quality, leaving the etiology of pneumonia unknown in about 50% of cases.^1^, ^2^, ^3^ Sputum contains a mixture of upper and LRT material, which impairs interpretation. Bronchoalveolar lavage (BAL) provides a high-quality LRT specimen, but it is invasive, costly, and not readily available.^4^ Viruses detected in upper respiratory tract specimens (eg, COVID-19, influenza virus, respiratory syncytial virus) may be responsible for LRT disease, but their isolation does not rule out other pathogens. Thus, improved ways to diagnose and characterize pneumonia that can help guide treatment and inform our understanding of disease pathogenesis are needed.^5^

We assessed whether PneumoniaCheck (MD Innovate, Inc), a device to collect coughed droplets onto a filter, could provide a noninvasive, easy-to-use way to collect a sample from the lung.^6^^,^^7^ Our group has previously demonstrated that PneumoniaCheck can detect bacterial DNA from the LRT.^6^, ^7^, ^8^ In this study, we evaluated the detection of SARS-CoV RNA and lung biomarkers in PneumoniaCheck-collected specimens from patients who had recently tested polymerase chain reaction (PCR) positive for COVID-19 by nasopharyngeal swab (NPS).

## Methods

### Study Population

Patients testing positive for SARS-CoV-2 and aged > 18 years were recruited from the Emory Acute Respiratory Clinic between April 1, 2021, and December 31, 2021, under an Emory institutional review board-approved protocol (MODCR002-STUDY00000863). These patients were asked to participate in the study during an Emory Acute Respiratory Clinic visit at which time they were receiving monoclonal antibody infusions.^9^ These patients had an NPS specimen positive for COVID-19 within 7 days of their study visit and met eligibility criteria for monoclonal antibody infusion (e-Table 1). After written informed consent, patients provided coughed specimens during a 1- to 2-hour infusion and had their electronic medical records reviewed, and demographics, clinical history, and outcomes abstracted. Control specimens delinked from personal identifiers and without other information were obtained after written informed consent from healthy adults between 18 and 45 years of age from April 1, 2021, to September 1, 2022.

### Outcomes of Interest

Our primary outcome of interest was the sensitivity and specificity of detecting SARS-CoV-2 RNA in cough compared with NPS specimen and specimen quality (ie, lower vs upper respiratory origin). Next, we examined inflammatory biomarker levels in case and control specimens. We examined associations between average cycle threshold (Ct) values and clinical, demographic, and prior vaccination characteristics. Finally, we assessed associations between biomarker levels and SARS-CoV-2 RNA and clinical features of the patient’s illness.

### Specimens

#### Specimen Collection

Cough droplet samples were collected using the PneumoniaCheck device (e-Fig 1). Patients were instructed to breathe deeply, cough into the device, and then exhale to end expiration while maintaining a tight seal around the mouthpiece of the device. Each participant tried 5 rounds of 10 coughs at 20- to 30-minute intervals for a total of 50 coughs.

#### Specimen Processing

The cough device was kept at 4 °C after collection until it was stored at −80 °C. The device was processed after acclimatizing to room temperature for 30 minutes in a Class II Biological Safety Cabinet (The Baker Company or Nuaire) using enhanced BSL2+ biologic safety personal protective equipment precautions. The filter was removed from the device, cut in one-half with a decontaminated, sterilized scissor, and one-half was placed in an Eppendorf tube containing 500 μL of RNAZol RT (Sigma Aldrich) for PCR studies. The other one-half was placed in an Eppendorf tube containing 300 μL of NP40 lysis elution buffer (diluted 1:1 with sterile phospate-buffered saline to achieve 0.5% NP40) (Invitrogen) for biomarker and other studies. The tube for biomarker and other studies was incubated for 20 minutes to inactivate the virus. Residual fluid in the membrane was collected by centrifuging the membrane in a Spin-X column (Corning) at 10,000 rpm for 10 minutes (Beckman Coulter Microfuge 18 Centrifuge), adding the fluid at the bottom of the column to the fluid in the original tube and storing at −80 °C.

#### RNA Extraction

RNA extraction was carried out using an RNeasy Kit (QIAGEN) according to the manufacturer’s instructions. While in a chemical fume hood, 7 μL (1% of total volume) of BAN (Molecular Research Center, Inc) was added, the mixture was briefly vortexed for 10 to 15 seconds and then was incubated for 10 minutes. After incubation, the specimen was centrifuged at 12,000 rpm for 5 minutes. The colorless upper layer was transferred to an Eppendorf tube. Then 5 μL of RNA carrier (1 μg/μL) from QIAamp viral RNA mini kit (QIAGEN) and 600 μL of 100% ethanol were added and the tube was briefly vortexed. The RNA was purified using the QIAamp viral RNA mini kit following the manufacturer’s instructions. RNA was eluted from the kit’s column in 60 μL of elution buffer.

#### Reverse Transcription PCR

Each PCR reaction was performed with 5 μL of extracted RNA mixed with 5 μL of 4× reverse transcription PCR master mix buffer (Life Technologies), 1.5 μL of specific primers and probe (N1, N2, or E), and 8.5 μL of water. The reverse transcriptase reaction was run at 50 °C for 15 minutes, followed by 95 °C for 10 minutes. Amplification was set up at 95 °C for 15 seconds and 60 °C for 1 minute for a total of 40 cycles. Two SARS-CoV-2 TaqMan real-time reverse transcription PCR assays targeting the nucleocapsid gene (N1 and N2)^10^^,^^11^ and one targeting the envelope gene^12^ were used (Integrated DNA Technologies). All primers and TaqMan probes (2019-nCoV_N1, 2019-nCoV_N2, E gene assay first-line screening) and positive controls, which were used at 200 copies/reaction, were purchased from Integrated DNA Technologies.

#### Specimen Quality

We tested for amylase activity to indicate upper respiratory tract contamination and surfactant A levels to indicate lower respiratory tract origin of the specimen. Surfactant A was not detected in any specimens, and only amylase activity was considered for specimen quality.

#### Measurement of Proinflammatory Panel

A 50-μL aliquot of the coughed specimen eluted from the filter was tested without further dilution with the MesoScaleDiscovery Proinflammatory V-Plex human Panel 1 plate in the MESO QuickPlex SQ 120 (Meso Scale Diagnostics). This kit detects interferon gamma, IL-1β, IL-2, IL-4, IL-6, IL-8, IL-10, IL-12p70, IL-13, and tumor necrosis factor alpha (TNF-α). We followed the manufacturer’s instructions for the kit except for extending the standard curve by diluting 5-fold (200 μL + 800 μL diluent 2) the kit calibrator 1 solution followed by the recommended serial 4-fold dilutions to generate calibrators 2 through 7. This made it possible to estimate lower levels of analyte. With the limited specimen volume and low concentration of analyte, each specimen was measured once. We used the mean ± 3 SDs of no specimen control as the lower limit of detection (LLOD), and values less than the LLOD were assigned the value of one-half the LLOD for analysis.

### Statistical Analysis

Data are presented as median (interquartile range [IQR]), or number positive/number tested (%). The reported Ct values are the average between the probes 2019-cCoV_N1 and 2019-nCoV_N2. A Ct value of 40 was used as the upper limit of detection for all PCR assays to determine the relative quantity of SARS-CoV-2 in each specimen. A specimen was considered positive if 2 or 3 of the 3 primer pairs were positive, indeterminate if only 1 primer pair was positive, and negative if none were positive. The association between test results and categorical variables was assessed using Fisher exact test, and continuous variables were compared using the Wilcoxon rank sum test. Biomarker values were standardized by subtracting the mean of the variable from raw values and dividing it by 1 SD for univariate and multivariable modeling. Age, BMI, diabetes mellitus, and hypertension were controlled in all models to avoid potential confounding effects. The associations between clinical symptoms and inflammatory biomarker level, Ct value, or test results were analyzed using univariate logistic regression models. Negative binomial modeling was used to evaluate the relationship between symptom counts and inflammatory biomarker levels. A 2-sided *P* < .05 was considered statistically significant. Data were analyzed using SPSS Statistics version 28.0 (IBM Corp), SAS statistical software version 9.4 (SAS Institute Inc), and RStudio version 4.0.3 (The Comprehensive R Archive Network).

## Results

### Baseline Characteristics

We enrolled 50 patients who tested positive for SARS-CoV-2 and were referred for monoclonal antibody therapy and 18 healthy adult control patients without an acute respiratory illness. Six of the case patients were removed from the analysis: 2 for incomplete collection due to respiratory discomfort, 1 for to poor sample quality, 1 for lost sample, and 2 for symptoms not being recorded in their medical record. One of the 18 control specimens was excluded because they had a higher level of amylase activity and much higher levels of biomarkers than other specimens, indicative of significant upper respiratory tract contamination. The final analytical cohort consisted of 44 case patients and 17 control patients (Table 1). Fifty-four percent were female (54.5%), 50% were White, and the median age was 53 years (IQR, 40-63). The most common comorbidities were hypertension (36.4%), diabetes (20.5%), and chronic kidney disease (11.4%). The most common reported symptoms were cough (84.1%), sinus congestion (72.7%), and fatigue (52.3%). Over one-half of the cohort was vaccinated before their study visit (65.9%) at a median time of 178 days (IQR, 130-209) after the second vaccine shot. Most vaccinated patients received the Pfizer vaccine (66.7%). Only 1 patient was hospitalized after their monoclonal antibody infusion for a COVID-19-related illness. This patient was hospitalized for 5 days and received steroids and anticoagulation.

**Table 1:** Characteristics of Case Patients

Characteristic	Analytical Cohort (n = 44)
Demographics	
Age, y	53 (40-63)
Sex	
Male	20 (45.5)
Female	24 (54.5)
Race	
White	22 (50.0)
Black	12 (27.3)
Hispanic	2 (4.5)
Asian	1 (2.3)
Unknown	7 (15.9)
BMI, kg/m^2^	31.6 (26.3-37.1)
Clinical history	
Diabetes mellitus	9 (20.5)
Hypertension	16 (36.4)
Tobacco use	0 (0)
Malignancy	5 (11.4)
Stroke/TIA	0 (0)
Chronic kidney disease	5 (11.4)
COVID-19 symptoms	
Time from symptom onset to positive test, d	2 (1-3)
Fever	17 (38.6)
Sore throat	10 (22.7)
Chills	14 (31.8)
Body aches	22 (50.0)
Sinus congestion	32 (72.7)
Loss of smell/taste	19 (43.2)
Cough	37 (84.1)
Shortness of breath at rest	6 (13.6)
Shortness of breath on exertion	15 (34.1)
Wheezing	6 (13.6)
Chest tightness	10 (22.7)
Confusion	4 (9.1)
Dizziness with standing	5 (11.4)
Headache	17 (38.6)
Diarrhea	13 (29.5)
Abdominal pain	5 (11.4)
Nausea	8 (18.2)
Rash	2 (4.5)
Joint pain	6 (13.6)
Fatigue	23 (52.3)
Weakness	11 (25.0)
Sleep disruption	10 (22.7)
Palpitations	3 (6.8)
Sweats	8 (18.2)
Anorexia	10 (22.7)
Anxiety	2 (4.5)
Vaccination history	
Vaccinated before positive test	29 (65.9)
Days from second vaccine shot to positive PCR test	178 (130-209)
Vaccine brand received	
Pfizer	18 (66.7)
Moderna	6 (22.2)
J&J	2 (7.4)
Combination	1 (3.7)

Data are presented as median (interquartile range) or No. (%). PCR = polymerase chain reaction; TIA = transient ischemic attack.

### Specimen Quality

Thirty-nine of 44 case specimens (89%) and 15 of 17 control specimens (88%) had undetectable levels of amylase activity. Amylase activity ranged from 3.2 to 43.4 mU/mL in the 5 amylase-positive case specimens and 8.7 to 82.3 mU/mL in the 2 amylase-positive control specimens. The specimens were collected with a median of 4 days (IQR, 3-5.25 days) after the PCR-positive NPS specimen.

### SARS-CoV-2 PCR Positivity

Of the 44 case patients, 30 (68%) tested positive for SARS-CoV-2 RNA in their coughed specimen (Table 2). The median PCR Ct value was 32 (IQR, 30-35). No significant differences were found in sex, race, clinical history and comorbidities, and time from symptom onset to cough sample collection between individuals who tested positive and those who tested negative.

**Table 2:** Comparison of Case Patients by Cough Specimen SARS-CoV-2 PCR Positivity

Variable	Test Status^a^			*P* Value
	Positive (n = 30; 68%)	Negative (n = 10; 23%)	Indeterminate (n = 4; 9%)	
Days from positive nasopharyngeal swab to cough sample collection	4 (3-6)	5 (2-5.5)	4 (2.25-5.75)	.90
Days from symptom onset to cough sample collection	5 (4-7)	7 (4-9)	7 (6.25-7.75)	.30
Sex				.10
Female	14 (47)	6 (60)	4 (100)	
Male	16 (53)	4 (40)	0 (0)	
BMI, kg/m^2^	32.5 (28-38.2)	28.5 (26.1-36.8)	26 (22.9-34.4)	.20
Race				.10
Asian	1 (3.3)	0 (0)	0 (0)	
Black	6 (20)	5 (50)	1 (25)	
Hispanic	0 (0)	2 (20)	0 (0)	
White	18 (60)	2 (20)	2 (50)	
Unknown	5 (16.7)	1 (10)	1 (25)	
Diabetes mellitus	6 (20)	3 (30)	0 (0)	.50
Hypertension	11 (36.7)	5 (50)	0 (0)	.20
Cancer	4 (13.3)	1 (10)	0 (0)	.70
Chronic kidney disease	3 (10)	2 (20)	0 (0)	.50

Data are presented as median (interquartile range) or No. (%). Wilcoxon rank sum test was used to compare continuous variables, and Fisher exact test was used to compare categorical variables. PCR = polymerase chain reaction.

a Test status is for SARS-CoV-2 RNA PCR on cough specimen. All case patients were SARS-CoV-2 RNA PCR positive on an earlier upper respiratory tract specimen.

#### Inflammatory Biomarkers

Because the levels of IFN-γ were below the LLOD for 91% of cases and 76% of control patients, we did not include it in the biomarker analyses. As indicated in Table 3, levels of IL-13 and TNF-α were higher (*P* = .001 and *P* = .03, respectively) and IL-2 lower (*P* < .001) in cases compared with control cough specimens. Within the case specimens, IL-1β and IL-8 levels were higher in PCR-positive patients than in PCR-negative patients (*P* = .03 and *P* = .02, respectively) (Table 4).

**Table 3:** Median Inflammatory Biomarker Levels Stratified by COVID-19 Cases and Control Participants

Biomarkers	LLOD	Cases (n = 44)	Cases %<LLOD, %	Control Participants (n = 17)	Control Participa nts %<LLOD, %	*P* Value
IL-10	0.028	0.028 (0.014-0.033)	45	0.031 (0.029-0.035)	24	.20
IL-12p70	0.035	0.088 (0.077-0.096)	0	0.079 (0.051-0.098)	6	.20
IL-13	0.039	0.62 (0.56-0.65)	0	0.33 (0.23-0.57)	0	.001
IL-1β	0.029	0.14 (0.12-0.18)	0	0.13 (0.11-0.14)	0	.10
IL-2	0.088	0.16 (0.13-0.18)	16	0.22 (0.20-0.23)	0	< .001
IL-4	0.007	0.009 (0.008-0.013)	23	0.008 (0.004-0.009)	29	.20
IL-6	0.059	0.08 (0.07-0.11)	14	0.07 (0.03-0.10)	47	.20
IL-8	0.01	0.13 (0.05-0.94)	0	0.09 (0.06-0.38)	0	.40
TNF-α	0.013	0.027 (0.015-0.050)	23	0.016 (0.007-0.022)	35	.03

Values are in pg/mL and presented as median (interquartile range) or as otherwise indicated. Wilcoxon rank sum test was used to compare variables. %<LLOD = percent of patient specimens with values below the LLOD; LLOD = lower limit of detection; TNF-α = tumor necrosis factor alpha.

**Table 4:** Comparison of Case Patient Biomarker Levels Test Status

Inflammatory Biomarker	Lower Limit of Detection	Indeterminate (n = 4; 9%)	Test Positive (n = 30; 68%)	Status Negative (n = 10; 23%)	*P* Value^a^
IL-10	0.028	0.033 (0.027-0.041)	0.021 (0.014-0.030)	0.028 (0.014-0.052)	.40
IL-12p70	0.035	0.076 (0.074-0.082)	0.088 (0.076-0.096)	0.094 (0.083-0.118)	.20
IL-13	0.039	0.61 (0.59-0.63)	0.61 (0.55-0.065)	0.64 (0.56-0.67)	.70
IL-1β	0.029	0.13 (0.12-0.15)	0.15 (0.13-0.20)	0.12 (0.10-0.13)	.027
IL-2	0.088	0.18 (0.14-0.19)	0.15 (0.13-0.18)	0.17 (0.10-0.20)	.60
IL-4	0.007	0.011 (0.008-0.016)	0.008 (0.004-0.012)	0.009 (0.009-0.018)	.10
IL-6	0.059	0.08 (0.06-0.09)	0.08 (0.07-1.67)	0.07 (0.04-0.08)	.10
IL-8	0.01	0.05 (0.04-0.05)	0.33 (0.07-1.67)	0.09 (0.04-0.20)	.024
TNF-α	0.013	0.021 (0.017-0.024)	0.030 (0.021-0.051)	0.013 (0.007-0.051)	.30

Values are pg/mL and presented as median (interquartile range) or as otherwise indicated. Wilcoxon rank sum test was used to compare values between groups. TNF-α = tumor necrosis factor alpha.

a *P* value represents comparison of values for patients who tested SARS-CoV-2 positive compared with those that tested negative and does not include control participants or indeterminate patients.

### Association Between Ct Values, Inflammatory Biomarkers, and Symptom Presence

No significant associations were found between Ct value and symptoms in univariate and multivariable models. Among reported symptoms, only fever and fatigue were significantly associated with inflammatory biomarker levels (Table 5). Higher IL-10 values were associated with reduced fatigue in multivariable analysis (OR, 0.41; 95% CI, 0.15-0.87; *P* = .039). Higher IL-12p70 (OR, 2.74; 95% CI, 1.15-8.51), IL-4 (OR, 3.5; 95% CI, 1.56-11.20), and TNF-α (OR, 4.36; 95% CI, 1.79-14.60) values were significantly associated with fever in the multivariable analysis.

**Table 5:** Multivariable Models for the Association Between Cycle Threshold Value, Inflammatory Biomarker Values, Fatigue, and Fever

Biomarker	Fatigue		Fever	
	Adjusted OR^a^ (95% CI)	*P* Value^a^	Adjusted OR^a^ (95% CI)	*P* Value^a^
IL-10	0.41 (0.15-0.87)	.039	2.18 (1.03-5.19)	.05
IL-12p70	0.91 (0.47-1.72)	.80	2.74 (1.15-8.51)	.043
IL-13	1.17 (0.61-2.30)	.60	1.23 (0.61-2.63)	.60
IL-1β	0.72 (0.33-1.37)	.30	1.13 (0.50-2.27)	.70
IL-2	1.85 (0.97-3.90)	.10	0.62 (0.27-1.27)	.20
IL-4	0.46 (0.17-0.93)	.06	3.56 (1.56-11.20)	.008
IL-6	1.26 (0.67-3.00)	.50	2.04 (0.86-9.54)	.20
IL-8	0.64 (0.24-1.25)	.20	1.51 (0.73-3.47)	.30
TNF-α	0.59 (0.28-1.15)	.10	4.36 (1.79-14.60)	.004
Cycle threshold	1.63 (0.76-3.79)	.20	0.47 (0.16-1.12)	.10

TNF-α = tumor necrosis factor alpha.

a Models were adjusted for age (years), BMI (kg/m^2^), diabetes, and hypertension. *P* < .05 was considered significant.

### Association Between Inflammatory Biomarkers and the Number of Symptoms

The adjusted negative binomial model demonstrated no significant association between biomarker levels and number of symptoms (e-Table 2).

## Discussion

In this study, specimens collected with PneumoniaCheck had (1) PCR-detectable SARS-CoV RNA, (2) detectable levels of inflammatory biomarkers, (3) little or no contamination from the upper respiratory tract, and (4) significant differences in some biomarkers between case and control specimens and different categories of case patient specimens.

Our laboratory previously showed that PneumoniaCheck specimens from the lower respiratory tract of patients with cystic fibrosis contained datable bacterial DNA.^7^^,^^8^ Cough specimens are less invasive than other lower respiratory tract collected specimens (eg, BAL) and can be collected in ambulatory settings.^7^^,^^8^ In a study by Patrucco et al^13^ of 12 patients with presumed viral pneumonia, viruses were detected by PCR in PneumoniaCheck specimens with a sensitivity of 66% compared with BAL. This level of sensitivity is similar to our detection rate of 68% in patients with an NPS specimen positive for SARS-CoV-2 RNA collected at a median of 4 days earlier. Ideally, specimens would be collected simultaneously, which may have improved the sensitivity of the PneumoniaCheck compared to NPS specimens. There was no evident change in detection rates during the study and change in circulating strains did not affect the PCR assays. The lower sensitivity of the PneumoniaCheck relative to NPS suggests its greatest value will be to understand the risk of transmission, the relationship between lung virus titer and disease, and the detection of biomarkers to understand the status and pathogenesis of lung disease rather than for diagnosis. Others described detecting SARS-CoV-2 RNA by PCR in aerosol specimens from NPS-positive patients including 1 of 17 exhaled breath condensate specimens,^14^ 13 of 29 modified N-95 mask collected specimens,^15^ and 36 of 48 face mask sampling collected specimens.^16^

The ability to detect biomarkers in the coughed droplets captured by PneumoniaCheck suggests this specimen can inform disease in the lung. Coughs generate droplets that are larger in size and volume than breathing-generated droplets, and they represent a sample of the lower respiratory tract secretions that may include secretions from alveoli.^17^, ^18^, ^19^ Disease in the lower respiratory tract has been a common feature of and important to the pathogenesis of COVID-19 disease.^20^ The low or negative amylase activity levels suggests minimal upper respiratory tract contamination in PneumoniaCheck specimen, supporting their lower respiratory tract origin and view of disease in the lung as previously noted.^6^, ^7^, ^8^

Because case patients were receiving highly effective monoclonal antibody infusions, we could not link PCR Ct values and biomarker detection to the course of the disease. Other biomarker studies most often have tested sera from patients with COVID-19.^21^, ^22^, ^23^ Some of these biomarker studies have suggested inflammatory cytokines and interferons have a positive feedback loop, resulting in a cytokine storm that is associated with vascular damage and organ dysfunction.^24^^,^^25^ Tocilizumab, a recombinant monoclonal antibody that inhibits IL-6, has been used to treat the cytokine storm associated with severe disease and is associated with improved mortality in critically ill patients.^26^, ^27^, ^28^, ^29^ A variety of other associations between biomarkers and COVID-19 have been described, including a dose-response relationship between the presence of certain cytokines and disease severity^30^ and NLRP3 inflammasome initiation during COVID-19, leading to increased production of proinflammatory IL-18 and IL-1β. The induction of IL-18 and IL-1 has been correlated with disease severity.^31^ In a study of nonhospitalized patients with mild to moderate COVID-19, a variety of increased serum cytokine levels compared with levels in control patients including IL-13 were described.^31^

There is less information about inflammatory biomarkers in the lung. The studies of lung biomarkers have usually been with BAL specimens in hospitalized patients with severe disease and not in nonhospitalized patients with the mild to moderate disease described in this study. In 1 study of hospitalized patients with severe COVID-19, investigators compared biomarker levels in the blood vs BAL specimens collected in parallel.^32^ They found higher levels in BAL compared with blood for some biomarkers (eg, IL-8, IL-1β) and lower levels for other biomarkers (eg, IL-2, IL-10, TNF-α) in patients with COVID-19 infection in intensive care units. The differences between biomarkers in BAL compared with blood is not surprising and illustrates the added value of getting specimens from the lung, an important site of COVID-19 disease. In the case vs control comparison, we found increased IL-13 and TNF-α and decreased IL-2 levels among cases compared with control patients. Among cases, we found increased IL-1β and IL-8 levels in PCR-positive cough specimens compared with PCR-negative cough specimens and increased IL-10 levels to be associated with less fatigue. IL-12p70 showed an association with fever. IL-10 is an antiinflammatory cytokine, and its role in COVID-19 infection has been largely undefined. Queiroz et al^33^ found that IL-10 levels were likely to be lower in patients with long-COVID compared with acute infections and may correspond with fewer infectious sequelae. On the other hand, IL-12p70 is a proinflammatory cytokine; however, its role in COVID-19 severity and progression has varied among studies.^34^^,^^35^ Quantifying these biomarkers in cough samples using the PneumoniaCheck device may help track disease progression and severity, both acutely and long-term. It will be important to confirm findings in this study, and in future studies, useful to have a larger number of control patients that are age-matched and compare results for COVID-19 with other respiratory infections and lung diseases. It would also be of interest to simultaneously compare biomarkers in cough specimens with those in blood.

The device used in this study provides a noninvasive way to obtain a high-quality LRT specimen. The need for multiple cough collection sessions to get the sample for this study presents a challenge for collection. In this study, we took advantage of the time needed for the patient to receive monoclonal antibody infusion therapy to collect the specimens. It provided a window of time for the specimen collection. Importantly, once instructed and supervised for the first collection, most patients were able to independently perform the remaining collections and provide good specimens. It is likely this will be practical in other settings (eg, home, outpatient clinic, emergency department, hospital) and can be done multiple times to monitor the course of the lung disease. Because, as previously noted, the patients received highly effective monoclonal antibody infusion treatment, future study is needed to determine if PCR or biomarker results from the cough specimen are associated with disease progression and the kinetics of SARS-CoV-2 RNA and biomarkers during infection, how it might inform transmissibility, and what other biomarkers might be detectable in it. The fact that several biomarkers were at low levels or below the assay’s limit of detection (eg, surfactant A, interferon gamma) indicates a need for more sensitive assays.

Because our study was performed at a single center in the subset of patients with COVID-19 with symptoms and risk factors that led to referral for monoclonal antibody infusion therapy, future studies will determine how well our results indicate results with other patient groups and settings. For example, specimen quality (ie, amount of lower respiratory tract specimen collected with minimal upper respiratory tract contamination) largely depends on patient compliance and likely varies among patients and settings. Patient training and factors that affect patient compliance with collection methods (eg, severity of illness) will likely contribute to variation in specimen quality. The potential for specimen variation underlines the importance of testing specimens for amylase activity and surfactant levels or other measures to assess specimen quality. Unfortunately, the assay for surfactant A was not sufficiently sensitive and was not helpful in evaluating specimen quality.

### Interpretation

In conclusion, PneumoniaCheck was shown to be a noninvasive method to sample lower respiratory tract secretions usually with minimal upper respiratory tract contamination. The captured lower respiratory specimen can be used to detect COVID-19, other pathogens, and biomarkers to better understand COVID-19 and other lung diseases.

## Funding/Support

This work was supported by COVID-19 CURE awards, made possible by philanthropic support from the O. Wayne Rollins Foundation and the William Randolph Hearst Foundation.

## Financial/Nonfinancial Disclosures

The authors have reported to *CHEST Pulmonary* the following: L. J. A. is co-inventor on a patent for the PneumoniaCheck device through the Centers for Disease Control and Prevention, Atlanta, GA. D. N. K. is a co-inventor on the patent through the Georgia Institute of Technology, Atlanta, GA. E. J. A. was not at the time of the study but is now employed by Moderna, Inc. None declared (R. S. S., T.-C. C., D. S., B. H., H.-Y. S., S. J. J., B. R. R., L. A., B. A.).

## Supplementary Data

e-Online Data

e-Online Data

e-Online Data
