# Supplementary figures and images for: Detection of SARS-CoV-2 RNA and Biomarkers in Device-Captured Droplets From the Lung
Source: CHEST Pulm. 2025 Jan 22;3(3):100137. doi: 10.1016/j.chpulm.2025.100137 (PMC13418021; doi:10.1016/j.chpulm.2025.100137)

Supplemental Figure 1. PneumoniaCheckTM device instructions provided to study participants.

**
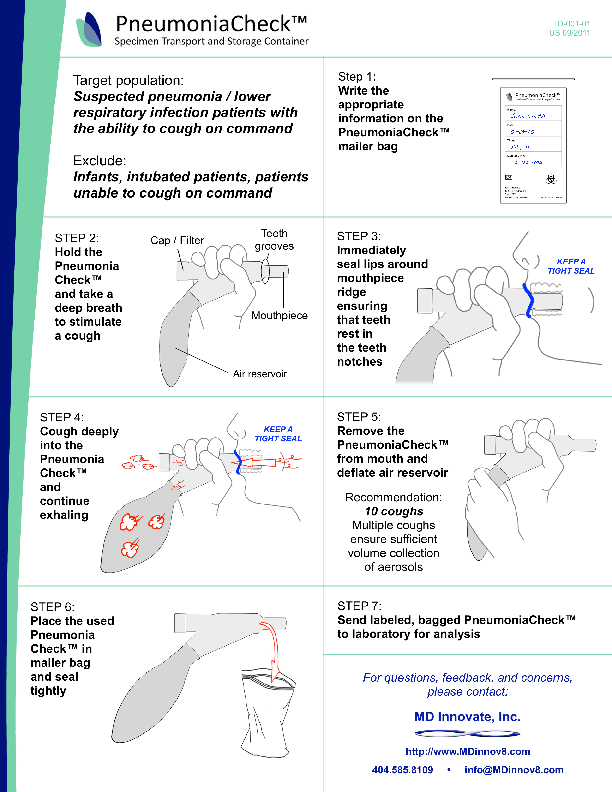
**

Supplement: e-Online Data [file mmc1.docx]
