# Supplementary material for: Detection of SARS-CoV-2 RNA and Biomarkers in Device-Captured Droplets From the Lung
Source: CHEST Pulm. 2025 Jan 22;3(3):100137. doi: 10.1016/j.chpulm.2025.100137 (PMC13418021; doi:10.1016/j.chpulm.2025.100137)
Supplement: e-Online Data [file mmc2.docx]

Supplemental Table 1. High-risk criteria for monoclonal antibody therapy infusion.

| mAB Infusion Criteria |
| --- |
| Aged >= 65 years |
| Obesity (BMI>30) |
| History of diabetes mellitus |
| History of cardiovascular disease (including congenital heart disease) or hypertension |
| History of chronic lung disease (e.g. chronic obstructive pulmonary disease, moderate-to-severe asthma, interstitial lung diases, cystic fibrosis, pulmonary hypertension) |
