# Supplementary material for: Detection of SARS-CoV-2 RNA and Biomarkers in Device-Captured Droplets From the Lung
Source: CHEST Pulm. 2025 Jan 22;3(3):100137. doi: 10.1016/j.chpulm.2025.100137 (PMC13418021; doi:10.1016/j.chpulm.2025.100137)
Supplement: e-Online Data [file mmc3.docx]

Supplemental Table 2. Negative binomial model for the association between symptom number and inflammatory biomarkers.

| Covariate | Percentage change in symptom counts (95% CI)* | P-Value |
| --- | --- | --- |
| Cycle Threshold | 1.02 (0.81-1.27) | 0.9 |
| IL-10 | 0.94 (0.73-1.21) | 0.6 |
| IL12p70 | 1.07 (0.82-1.40) | 0.6 |
| IL-13 | 1.01 (0.75-1.35) | 1.0 |
| IL-1β | 0.85 (0.67-1.06) | 0.1 |
| IL-2 | 1.07 (0.85-1.36) | 0.6 |
| IL-4 | 1.02 (0.81-1.28) | 0.9 |
| IL-6 | 1.08 (0.88-1.33) | 0.4 |
| IL-8 | 0.86 (0.68-1.06) | 0.2 |
| TNF-α | 1.12 (0.87-1.45) | 0.4 |

*The model adjusted for age (years), body mass index (kg/m^2^), diabetes, and hypertension.
